# Evaluation of self-educational training methods to learn laparoscopic skills - a randomized controlled trial

**DOI:** 10.1186/s12909-018-1193-3

**Published:** 2018-05-02

**Authors:** Steffen Axt, Pirmin Storz, Carolin Ehrenberg, Claudius Falch, Marc Immenroth, Andreas Kirschniak, Sven Muller

**Affiliations:** 1 Working Group for Surgical Technique and Training, Clinic for Visceral, General and Transplant Surgery, Tuebingen University Hospital, 72072 Tübingen, Germany; 2 Ethicon, Endo-Surgery Europe, Norderstedt, Germany

**Keywords:** Laparoscopic suturing and intracorporal knotting, Operation primer, Self-educational, Video-assisted learning

## Abstract

**Background:**

Evaluation of two different self-educational methods (video assisted learning versus video assisted learning plus a nodal point operation primer) on learning laparoscopic suturing and intracorporal knotting.

**Methods:**

Randomized controlled trial at the laparoscopic surgical training center, University of Tubingen with 45 surgical novices first year medical students being pretested for dexterity. After self-educational training for 90 min with either method (Group A: video assisted learning, Group B: video assisted learning plus a nodal point operation primer) participants had to perform five laparoscopic intracorporal knots. Assessed were number of knots completed (maximum of five knots counted, knot integrity, technical proficiency and knotting time per knot. Primary outcome measure is a composed knot score combining knot integrity, technical proficiency and knotting time.

**Results:**

Group B (*n* = 23) achieved a significantly higher composed knot score than Group A (*n* = 22) (53.3 ± 8.4 versus 46.5 ± 13.6 points respectively, *p* = 0.016). Median knotting time per completed knot was significantly different between Group B and Group A (308 s [100–1221] versus 394 s [138–1397] respectively, *p* = 0.001). Concerning number of completed knots there was a trend towards more knots achieved in Group B (4.2 ± 1.2 versus 3.55 ± 1.4 respectively, *p* = 0.075) .

**Conclusions:**

The use of a nodal point operation primer highlighting essential key steps of a procedure augment the success of learning laparoscopic skills as suturing and intracorporal knotting. (UIN researchregistry3866, March 22, 2018).

**Electronic supplementary material:**

The online version of this article (10.1186/s12909-018-1193-3) contains supplementary material, which is available to authorized users.

## Background

Learning laparoscopic surgical skills is a major challenge for interns and of paramount importance in any educational surgical residency program. Especially in the light of limited training opportunities in the operation room due to restricted working hours and operation costs at its limit, optimized resident teaching strategies are warranted [1]. Simulation based training methods have been published that support training success and transferability into the operation room [2]. However the instructional implementation strategies of simulation based resident training are less well investigated. Self-educational learning by video tutoring and following hands on training by the resident himself is one possible option [3]. An additional available teaching approach is “step-by-step”- instructions based on the philosophy of the “mental training”, repeatingly internalizing the essential key points of a procedure. Though, the actual mental training requires in the optimum case a “one-to-one” personal coaching and its practicability is therefor limited in everyday use.

The nodal point concept breaking down a procedure in a limited number of essential key steps significantly improves the learning and internalization of laparoscopic skills. This concept highlights crucial steps of a procedure and helps to repeatedly imagine the procedure [4].

The aim of this randomized controlled trial was to assess the value of an additional “nodal point operation primer” without the actual mental training in the process of video assisted self-educational learning of laparoscopic suturing and intracorporal knotting.

## Methods

### Setting and participants

This study was conducted by the Surgical Training Center at the University Tubingen. Because of the educational nature of the study, evaluation of the study protocol by the local ethics committee was not necessary. The participants were all first year medical students without pre-existing experiences in surgical techniques and especially no previous laparoscopic skills.

### Baseline assessment and pretests

In preparation all study participants received an instructional video to teach elementary basics of laparoscopy and laparoscopic suturing and intracorporal knotting. Afterwards, participants had to reply a standardized questionnaire about basic laparoscopy before study inclusion. Exclusion from the study was done if less than 60% of questions were correctly answered. After study inclusion two preliminary simulation based laparoscopic exercises and a laparoscopic intracorporal knot were performed in a black box trainer to assess baseline manual dexterity and the ability of three-dimensional orientation. For the first preliminary simulation based laparoscopic exercise participants had to touch 20 contact points of a 3-D model, in which every contact point had a diameter of 3 mm surrounded by a ring which could be illuminated by LEDs. Contacting the surrounding of the point was electronically detected as an error. For the second preliminary simulation based laparoscopic exercise the participants had to pass a needle horizontally through five openings in a wall with two grasping instruments. Touching the wall with the needle or the instruments was electronically detected as an error. In both exercises, the number of errors and the necessary performing time in seconds were determined. Finally the participants had to perform a pretest laparoscopic intracorporal knot in a maximum time span of seven minutes according to the below mentioned criteria after watching an uncommented video demonstrating laparoscopic suturing and intracorporal knotting.

### Randomization

After study inclusion and successful pretesting participants were randomly allocated to either group A (video assisted learning) or group B (video assisted learning plus operation primer) in a 1:1 ratio.

### Study performance

Participants of both groups received a standardized introduction to the laparoscopic workstation for 10 min. From now on Group A and B were separated for the learning phase and final testing.

### Group a – Video assisted learning

Participants of Group A watched a commented video of the performance of laparoscopic suturing and intracorporal knotting. Afterwards the video was shown during a training period of 90 min in a continuous loop so that a presentation of the performance was available for the participants all the time.

### Group B - video assisted learning plus a key point operation primer

Additionally to the video, the participants of Group B received an operation primer booklet highlighting the 10 key points of laparoscopic suturing and intracorporal knotting in text form and with example pictures (see Additional File 1). The concept of an operation primer is based on highlighting crucial steps of a procedure by key points and helps to repeatedly imagine the procedure. According to Immenroth et al. the definition of a key point is that it represents necessary structural motor components that must be performed in sequence and are marked by a reduction in the degrees of freedom of action (e.g correct alignement of the needle with the needle holder is a mandatory steps for a correct puncture of the wound edge in the following step) [4].

The correct use of the operation primer was shown in a standardized video presentation. Subsequently the participants had 90 min to train laparoscopic suturing and knots according to the video and the operation primer.

Finally after a 20 min break, each participant was instructed to perform five laparoscopic intracorporal knots in a maximum time span of 35 min without further help. The performance was video-taped for further evaluation.

### Outcome measure

The analysis of the videotapes and the knot specimen was performed independently by two blinded investigators (both board certified general surgeons). Assessed were the number of knots completed (maximum of five knots counted), knot integrity, technical proficiency and knotting time per knot.

The primary end point was a composed knot score combining knot integrity, the technical proficiency and the knotting time. Knot integrity was defined by a sufficient tightened and squared knot defined as, complete adaption of the wound margins (adaption of the sponge edged [knot not too loose]), correct distance of the needle injections, no tears in the sponge (knot too tight) as well as sufficient length of the rest of the suture (5 mm). A maximum of 6 points (one for each fulfilled evaluation criterion) could be reached for knot integrity.

Technical proficiency was defined as correctly performing the 10 key steps shown in the video and highlighted in the operation primer in the correct order. For reaching a step one could get two points. In addition, an extra-point was awarded for the right performance of the essential part of a step of the knotting technique. Thus, a maximum score of 30 points could be reached for technical proficiency.

Knotting time per knot was defined as time needed for the performance of one complete knot (knotting time) and was recorded in seconds. The knotting time was then transferred by means of a conversion table in a point value. The maximum achievable points given were 30 for a time needed of < 209 s for one knot. For each additional 35 s time needed a point was deducted.

As a maximum result a score of 66 points was possible. Only completely performed knots were evaluated for knot integrity, technical proficiency, knotting time and number of correctly performed knots.

The mentioned composed knot score used at the Surgical Training Center at the University Tubingen to compare the before/after results of laparoscopic knotting in the residency training program is similar to the knot score proposed by Aggarwal et al. [5].

### Materials

As suture material a 3–0 polyfilament suture with a 26 mm in diameter SH-needle (Ethicon Endo Surgery; Johnson and Johnson, New Brunswick; New Jersey, USA) was used. The length of the suture was standardized to 120 mm. The knots were performed on sponges with marks of 5 cm width and 1 cm height, which defined the area in which the knots had to be performed and in which a 8 cm long incision was placed in the center. The laparoscopic tower was equipped by Karl Storz endoscopes (Karl Storz Endoscopes GmbH and Co. KG, Tuttlingen, Germany). The equipment consisted of high density cameras (Karl Storz H3 Z Full-HD, Karl Storz SCB Image 1 HD Hub 222,010 20), 30°-optics (Karl Storz Hopkins®-II 30°-optics) and cold light sources (Karl Storz SCB xenon 300 20,133,120). The recordings were done by an integrated reception system (AIDA, Karl Storz Endoscopes GmbH and Co. KG, Tuttlingen, Germany). During the performance of the study knots, the optics were fixed in an angle of 45° by a Karl Storz Martins arm (Karl Storz Endoscopes GmbH and Co. KG, Tuttlingen, Germany). The used laparoscopic instruments were a needle holder (Karl Storz, needle holder to Koh Marco; straight grip), a laparoscopic atraumatic grasping forceps (Karl Storz; click-Line Dissecting and Grasping Forceps according to Kelly, pistol grip) and a laparoscopic scissors (Karl Storz, click shear line; pistol grip).

### Statistical evaluation

Sample size calculation was done assuming a difference for the primary end point between group A and group B of greater than 20% to be relevant. This difference could be detected with a two-sided significance level α = 0.05 and a power of 1 − β = 0.9, with a group size of 22 participants randomized to each group. The statistical evaluation was performed with SPSS 21 (SPSS Inc., Chicago, IL, USA). First the arithmetic means and their standard deviations or medians and their interquartile ranges were determined. In the analytical statistics first the determination of the normal distribution was done. Subsequently, the test of homogeneity of variance followed. Significance was tested by the student t-test for independent samples or the Mann-Whitney-U-test for independent samples. The study was registered at www.researchregistry.com (UIN researchregistry3866).

## Results

**Fig. 1:**
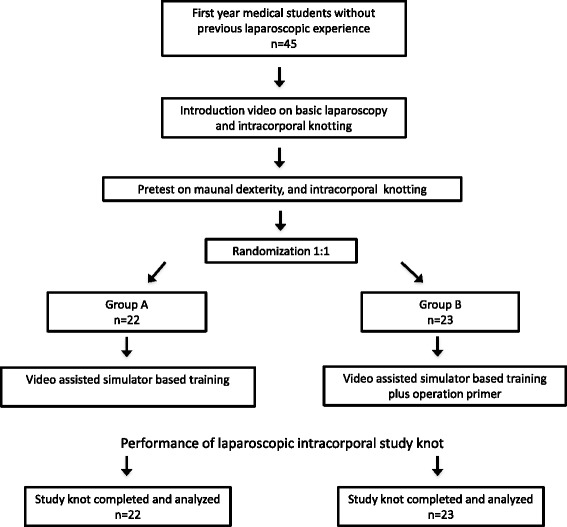
Study flow chart

Flow of participants through the study is displayed in Fig. 1. Participant characteristics and pretest results were well distributed and are shown in Table 1. None of the participants achieved to perform a complete pretest knot. Outcome measures are summarized in Table 2. For the primary outcome measure composed knot score group B (*n* = 23) achieved significantly more total points than group A (*n* = 22) (53.3 ± 8.4 versus 46.5 ± 13.6 points respectively, *p* = 0.016). Especially median knotting time per completed knot was significant different between Group B and Group A (308 s [100–1221] versus 394 s [138–1397] respectively, *p* = 0.001). Concerning number of completed knots there was a trend towards more knots achieved in Group B (4.2 ± 1.2 versus 3.55 ± 1.4 respectively, *p* = 0.075). Concerning the inter-rater reliability of the two blinded reviewers for the outcome measures, there was discordance in only one case judging tears in the sponge.

**Table 1:** Participant characteristics and pretest results

	Group A Video assisted learning	Group B Video assisted learning plus operation primer	*p*-value
N=	22	23	
Female Gender	10	16	0.11
Median age (years, [range])	21 [20–28]	21 [20–26]	0.71
Previous experience with video games (n=)	20	22	0.52
Right handed (n=)	19	18	0.48
Pretest manual dexterity [s]	100 [28–580]	107 [58–215]	0,42
Pretest three-dimensional orientation [s]	178 [62–404]	179 [83–475]	0,51
Pretest knot integrity points	1.17 ± 1.3	1.27 ± 1.1	0.78
Pretest technical proficiency points	8.7 ± 5.6	10 ± 6.4	0.48

**Table 2:** Participant outcome measures

	Group A Video assisted learning	Group B Video assisted learning + operation primer	*p*-value
n	22	23	
Total composed knot points	46.5 ± 13.6	53.3 ± 8.4	0.016
Knot integrity points	3.18 ± 1.2	3.4 ± 0.6	0.76
Technical proficiency points	22.7 ± 5.6	24.3 ± 2.9	0.22
Knotting time points	20.6 ± 8.6	25.5 ± 6.5	0.009
Mean Knotting time per knot (sec)	394 [138–1397]	308 [100–1221]	0.001
Number of correctly performed knots	3.55 ± 1.4	4.2 ± 1.2	0.075

## Discussion

The present trial shows that successful self-educational video assisted learning of laparoscopic skills with an additional operation primer highlighting the key points of the procedure can augment the trainees perfomance. Several studies have shown that simulation based laparoscopic training significantly improves the performance of residents and surgical beginners in the operation theatre [6, 7]. A main reason for the increasing number of available additional training tools is the financial pressure of hospitals that cannot offer surgical training any more exclusively in the operating room [1, 8]. However there are numerous training approaches for the acquisition of laparoscopic skills and their comparability and known effectiveness is limited [9]. A randomized controlled trial by Prabhu et al. showed that elevated stress level in the operating room resulted in an incomplete transfer of simulator acquired laparoscopic skills in daily practice [10]. Therefor it remains important to evaluate different training methods for their learning curves and effectiveness when implementing a laparoscopic training curriculum. Studies on autodidactic learning methods in laparoscopic simulator based training rarely exist. Xeroulis et al. compared in a randomized controlled trial computer based video instructions as a self educational method with expert feed back hands on training for laparoscopic knotting, showing no difference in learning success and learning curves for either method [11]. An additional available teaching approach is “step-by-step”- instructions based on the philosophy of the “mental training” [12]. Immenroth et al. described a significant difference in learning the laparoscopic cholecystectomy between practical and mental training [4]. Though, mental training requires in the optimum case a “one-to-one” personal coaching and its practicability is therefor limited in everyday use. Based on this principal of “step-by-step”- instructional mental training the concept of a nodal point operation primer divides and highlights a procedure in a limited number of essential key steps without the time and human resource consuming relaxation and repetition exercises. The aim of such a nodal point operation primer is a deeper and more structured internalization of the procedure by breaking it down to its essential 10 key points and therefore a higher and more sustainable chance of reproduction during a stressful situation [13]. Our results underline that for medical novices the acquisition of laparoscopic skills such as intracorporal suturing and knot tying is improved using an additional operation primer. Especially the time needed to perform a laparoscopic knot was significantly shorter and there was a trend towards more correctly performed knots in a predefined time period in the operation primer group.

A potential bias of this study is that during the practice period no data about the amount of time spent on the task of laparoscopic knotting and on the actual usage of the video and/or the operation primer are available. However this study was primarily designed to evaluate two self-educational methods of learning laparoscopic knotting accepting that the amount of resource used by each single participant might be different among the two groups.

Simulation based training irrespective of the method itself has been shown to have a positive effect on all aspects of laparoscopic learning and its transferability of the acquired skills to the operation room [14]. Akl et al. reported that by just showing an instructional and structured video to the participants before training a significant reduction of laparoscopic knotting time was achieved [15]. Further, limited evidence exists on the influence of different teaching methods used in simulator based laparoscopic training on long term skill retention. While repetitive hands-on training is shown to be more proficient, the amount of instructor feedback seems to have no impact long term laparoscopic skill retention [14, 16]. The effect of a nodal point operation timer on long-term skill retention has to be issue of further evaluation.

## Conclusion

Altogether, the use of a nodal point operation primer highlighting essential key steps of a procedure augments the success of performing laparoscopic skills as suturing and intracorporal knotting. It should therefor be implemented in self-educational simulation based laparoscopic teaching programs as it is a valuable tool in taking up a procedure.

## Additional file

Additional file 1: Institutional education booklet on laparoscopic suturing and knotting. An institutional education booklet explaining ten key steps of laparoscopic suturing and knotting. We have permission for publication of this institutional booklet from the University hospital Tuebingen, Germany. (PDF 1595 kb)
