# Supplementary material for: Evaluation of self-educational training methods to learn laparoscopic skills - a randomized controlled trial
Source: BMC Med Educ. 2018 May 2;18:85. doi: 10.1186/s12909-018-1193-3 (PMC5930485; doi:10.1186/s12909-018-1193-3)
Supplement: Supplementary file 1 — Institutional education booklet on laparoscopic suturing and knotting. An institutional education booklet explaining ten key steps of laparoscopic suturing and knotting. We have permission for publication of this institutional booklet from the University hospital Tuebingen, Germany. (PDF 1595 kb) [file 12909_2018_1193_MOESM1_ESM.pdf]

Techniques:
suturing and knot tying in laparoscopy

Minimal Invasive Chirurgie & Training – Arbeitsgruppe Experimentelle

Intracorporeal standard knot

1. Align the needle
2. Transfix the right edge of the wound
3. Pull the thread through
4. Transfix the left edge of the wound
5. Grasp the needlepoint
6. Wrap twice
7. Tie up the knot
8. Wrap once
9. Tie up the knot
10. Cut the thread

1. Align the needle

The left grasper holds the needle at the needlepoint. By giving the thread a tug just behind the needle, the needle can be aligned vertically. Now the needle holder takes the needle at the tail end.

The needle holder keeps the needle at a 90-degree angle to it`s shaft

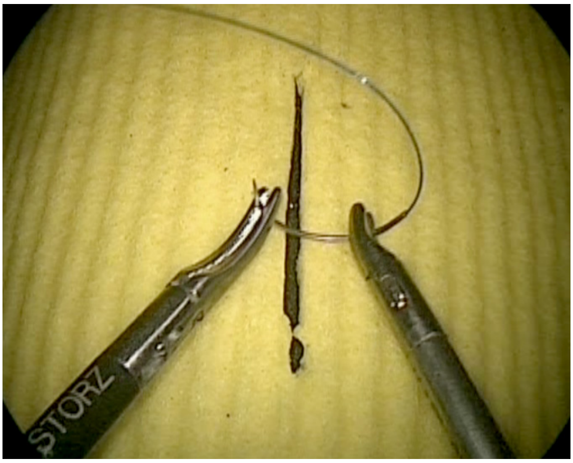

2. Transfix the right edge of the wound

Transfix the right wound edge with the needle. The puncture should be about 0.5 cm away from the wound edge. The stitch movement must be circular, so that the tissue is traumatized as little as possible. To hit the wound edge correctly, it can be raised with the left Grasper.

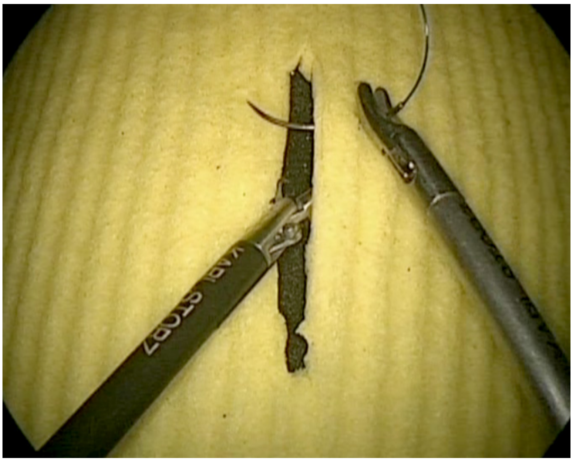

3. Pull the thread through

After the needle holder takes the needle at the tail end again the thread is pulled through the tissue up to 1-2cm. The left grasper can be used for tissue stabilization to avoid a traumatic tissue damage.

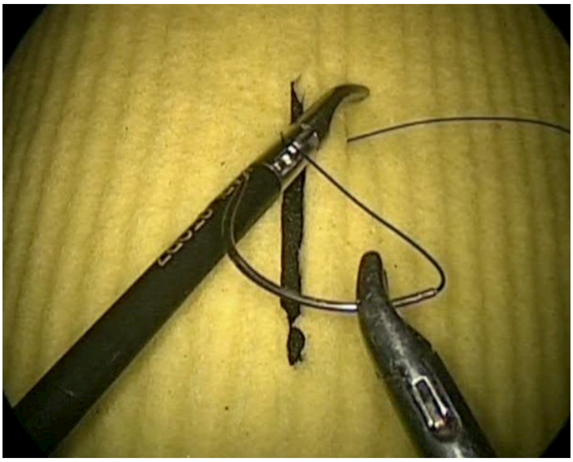

Transfix the left edge of the wound 4.

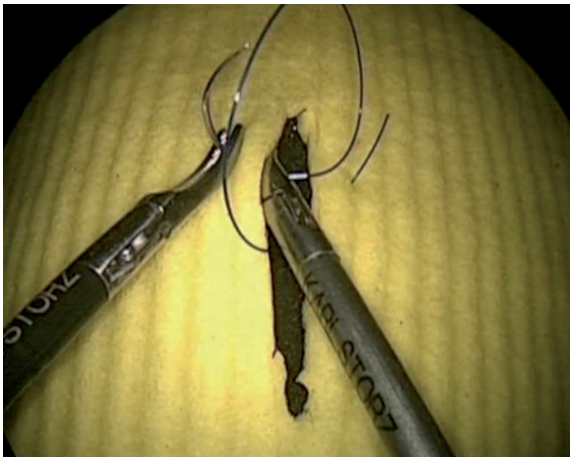

Now, transfix the left edge of the wound with the needle. Again the stitch movement must be circular and the extraction spot should be about 0.5cm away from the wound edge.

Grasp the needlepoint 5.

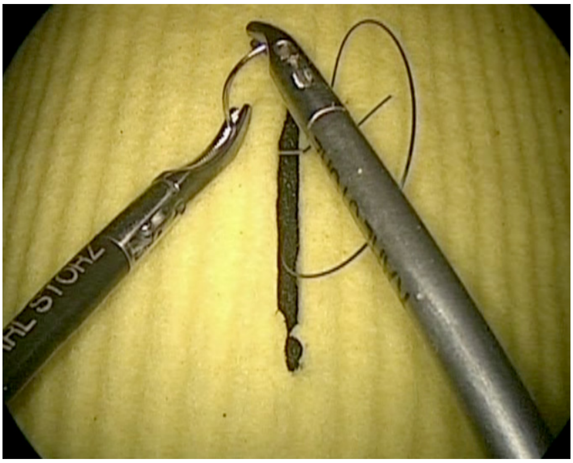

After puncturing the tissue the needle holder takes the needle at the needle-point.

Wrap twice 6.

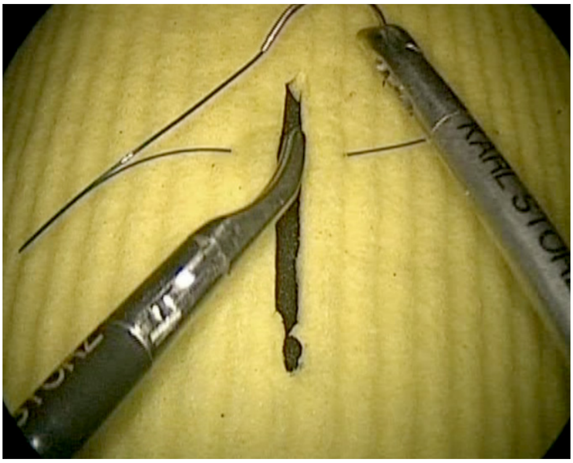

Wrap the thread twice the around the grasper. Thereby the thread must be wrapped from behind.

7. Tie up the knot

Grasp the end of the thread and tie up the knot. While tighten the knot pay attention that the engulfment are not lying on top of each other but are visible side by side.

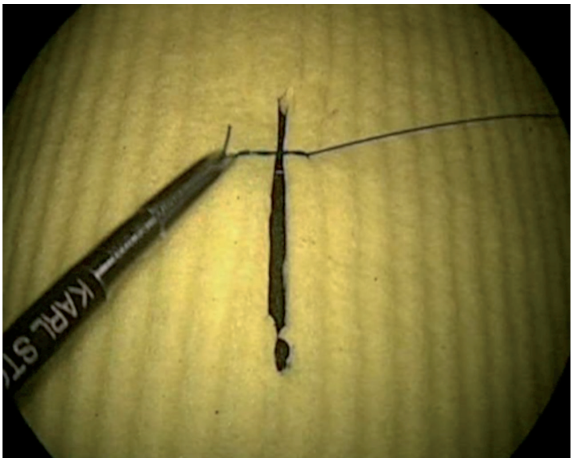

8. Wrap once

Before pulling the thread through the loop, make sure that two torsions are threaded. Pull the ends of the threat in a 90-degree angle to the wound edge.

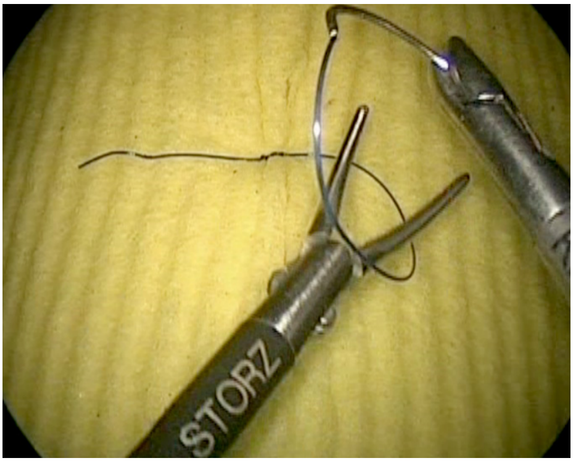

9. Tie up the knot

Tight up the knot again. When tightening, take care that the first knot doesn‘t loosen.

Repeat nodal points 8 and 9.

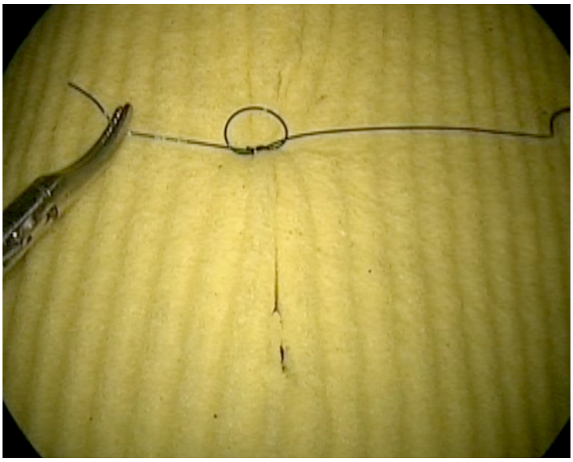

Cut the thread 10.

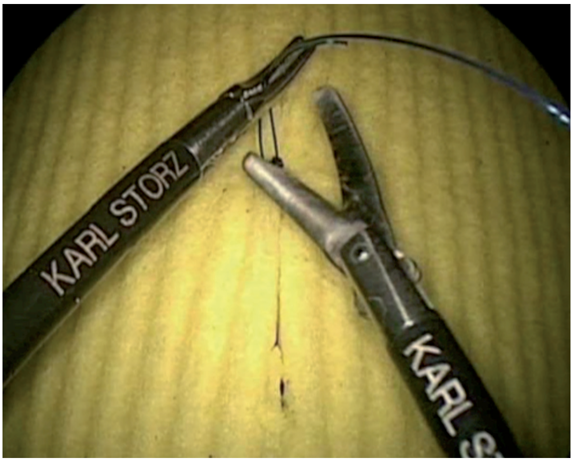

Finally, the thread is cut with a length of about 0.5cm.
